# Enhanced-view totally extraperitoneal repair in a patient with incisional hernia after robot-assisted laparoscopic radical prostatectomy: a case report

**DOI:** 10.1186/s40792-022-01380-2

**Published:** 2022-02-07

**Authors:** Shusaku Honma, Keisuke Tanino, Takashi Kumode, Ryosuke Mizuno, Yugo Matsui, Siyuan Yao, Teppei Murakami, Takatsugu Kan, Sanae Nakajima, Takehisa Harada

**Affiliations:** Department of Surgery, Kobe City Medical Center West Hospital, 2-4, Ichibancho, Nagataku, Kobe, Hyogo 653-0013 Japan

**Keywords:** Incisional hernia, eTEP, RARP, Ventral hernia

## Abstract

**Background:**

Although laparoscopic incisional hernia repair, especially laparoscopic intraperitoneal onlay mesh, is a widely used technique, it can cause serious complications, including mesh erosion, adhesive bowel obstruction, and chronic pain. The enhanced-view totally extraperitoneal (eTEP) technique has been reported to prevent such complications by placing the mesh in the retrorectus space. Here, we report the case of a patient with post-robot-assisted laparoscopic radical prostatectomy (RARP) incisional hernia repaired using the eTEP technique.

**Case presentation:**

A 67-year-old man, who underwent RARP for prostate cancer 4 years ago developed an incisional hernia. Abdominal computed tomography showed the presence of an epigastric incisional hernia measuring 4 cm long and 3.7 cm wide. We performed an eTEP repair. We closed the hernia defect using a 0 barbed suture and placed a self-gripping mesh measuring 20 cm long and 15 cm wide in the developed retrorectus space with no fixation. There were no postoperative complications, and the patient was discharged on postoperative day 2.

**Conclusions:**

eTEP repair is considered an extremely effective surgical treatment option for incisional hernias because of its few resulting postoperative mesh-and-tacker-related complications.

## Background (Introduction)

Recently, robot-assisted procedures have become widely used in various surgeries; in particular, robot-assisted laparoscopic radical prostatectomy (RARP) for prostate cancer has been one of the most accepted surgeries. RARP has clinical, functional, and oncological benefits in patients with prostate cancer [1, 2]. However, according to recent studies, the incidence rate of incisional hernia is higher with minimally invasive radical prostatectomy, including RARP, than with traditional open radical prostatectomy [3, 4].

Incisional hernia is one of the most common postoperative complications experienced by patients, which not only causes pain and cosmetic problems, but also causes incarceration of the small bowel [5]. Emergency surgery is necessary for the incisional hernia incarceration. Even without incarceration, surgical treatment is required to cure the incisional hernia, often leading to further physical and economic burdens on the patient [5].

The enhanced-view totally extraperitoneal (eTEP) technique, an endoscopically performed Rives–Stoppa technique, was first described by Miserez for ventral hernia repair [6]. The eTEP technique has been reported to be able to avoid common mesh-and-tacker-related complications from laparoscopic intraperitoneal onlay mesh (IPOM) technique, i.e., mesh erosion, adhesive bowel obstruction, and chronic pain, by placing the mesh in the retrorectus space [7].

To the best of our knowledge, no other study has reported a case of eTEP repair in a patient with incisional hernia after RARP. Here, we report a case of a patient with post-RARP incisional hernia repaired using the eTEP technique.

## Case presentation

**Fig. 1:**
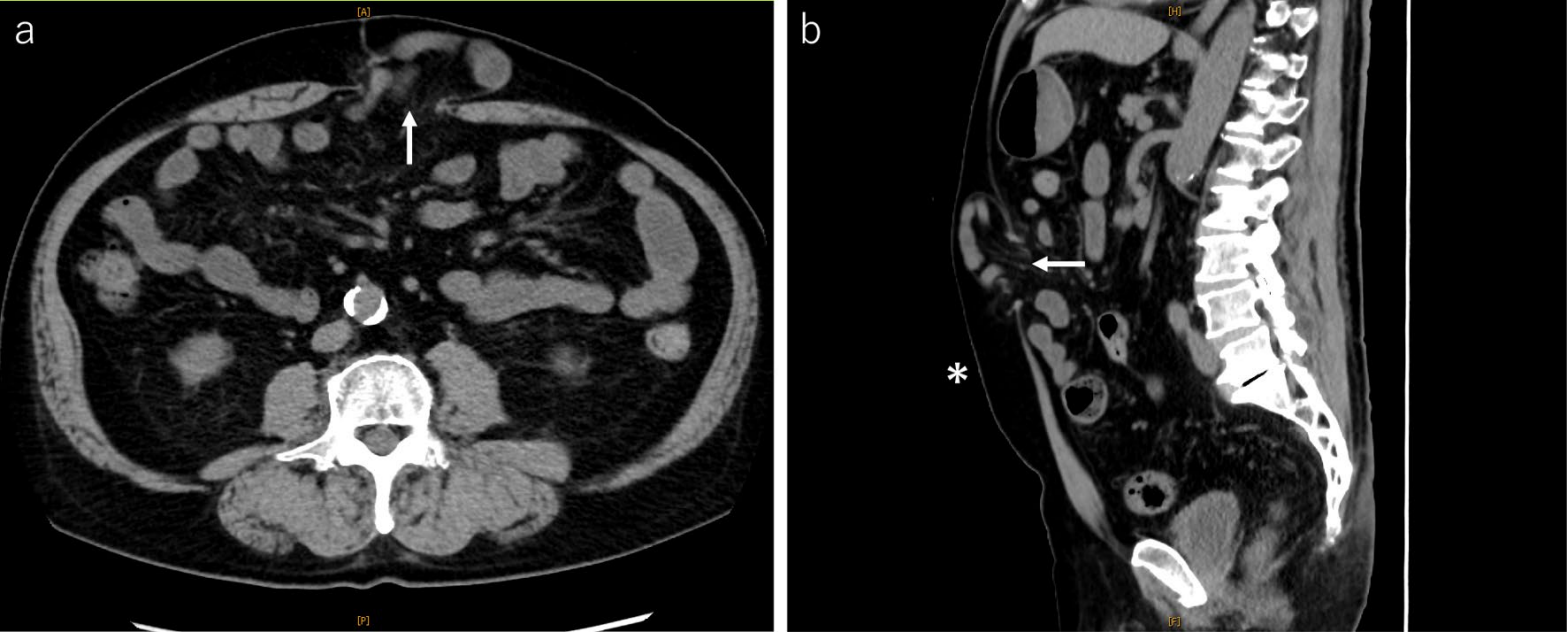
Abdominal computed tomography. Abdominal computed tomography demonstrates an incisional hernia orifice in the upper abdomen. **a** Axial image showing the hernia defect measuring 3.7 cm wide (arrow). **b** Sagittal image showing the hernia defect measuring 4.0 cm long (arrow). The defect is located in the upper umbilicus (*)

A 67-year-old man presented to our department with a swelling in the upper abdomen for half a year. His surgical history included RARP for prostate cancer in 2017. He had been medicated with a few anti-anxiety drugs for depressive anxiety disorder. Physical examination revealed a tennis ball-sized irreducible subcutaneous mass on the previous RARP incision scar, where the specimen was removed from the upper abdominal midline. Blood tests, including blood cell counts, liver enzymes, renal function, and HbA1c, were within the respective reference ranges. Abdominal computed tomography showed the presence of an epigastric incisional hernia 4 cm long and 3.7 cm wide (Fig. 1). We decided to perform an eTEP repair.

**Fig. 2:**
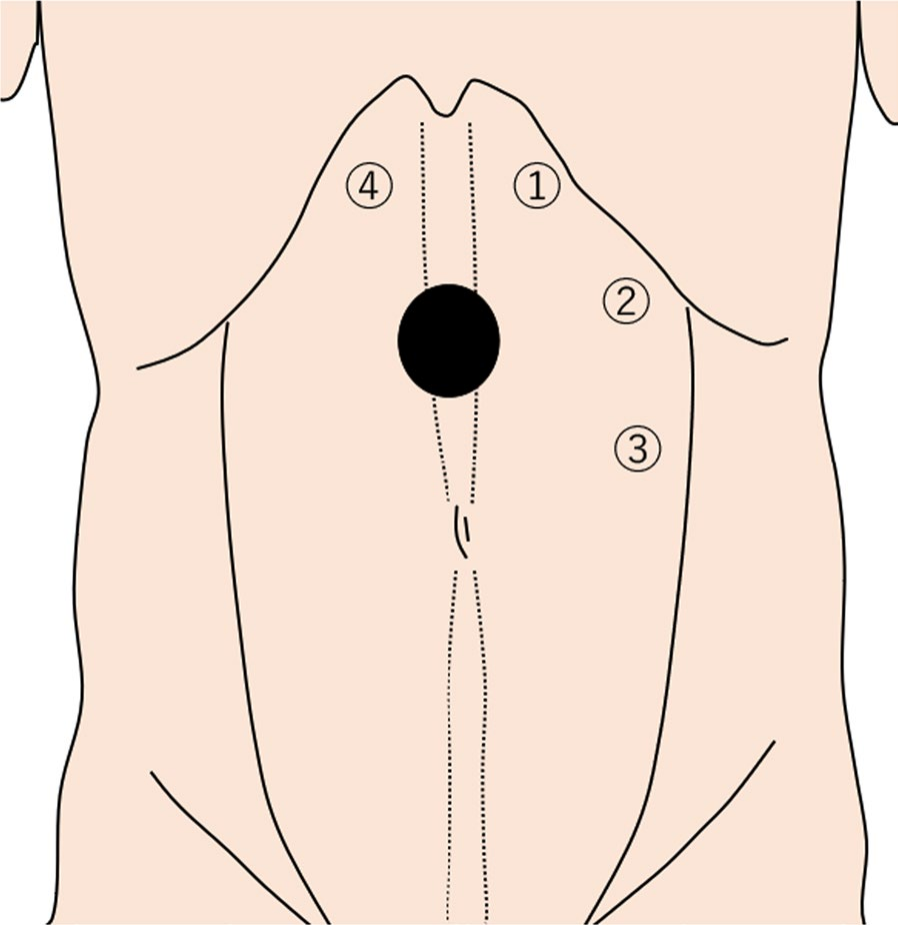
Schema of endoscopic ports placement. Schema of endoscopic port placement. The hernia defect was located in the epigastric area

The patient was placed in the supine position with his arms tucked at the sides under general anesthesia. Figure 2 shows the port placement in this case. We made a 2-cm skin incision at the port 1 position and identified the anterior rectus sheath, then incised it sharply. We used balloon dissector to develop the left retrorectus space after splitting the rectus abdominis muscle, inserted a 12-mm trocar, and performed pneumoperitoneum at 10 mmHg. Two 5-mm trocars were inserted at port 2 and port 3 positions under the endoscopic view.

**Fig. 3:**
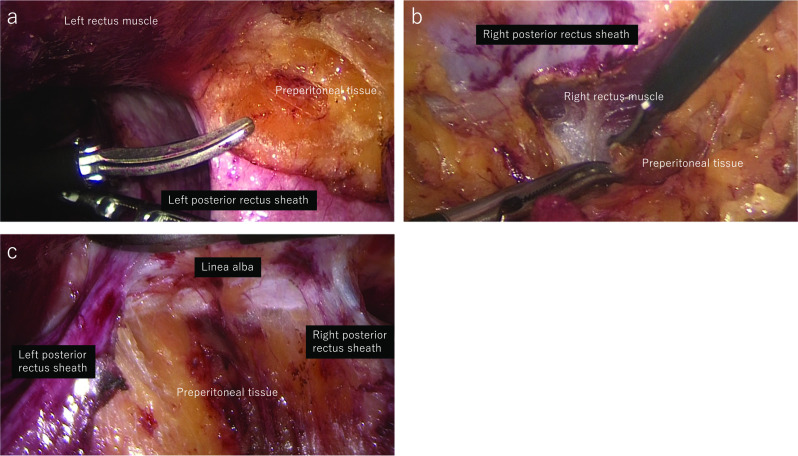
Intraoperative endoscopic views-1. Intraoperative endoscopic view. **a** Medial portion of the left posterior rectus sheath was incised. **b** Medial portion of the right posterior rectus sheath was incised after crossing the preperitoneal space. **c** Retrorectus space and preperitoneal space were dissected from the cephalad to caudal direction, incising bilateral posterior rectus sheaths

Above the level of the hernia defect, the medial portion of the left posterior rectus sheath was incised from the cephalad in the caudal direction and crossing to the preperitoneal space under the white line to the right posterior rectus sheath. The medial portion of the right posterior rectus sheath was incised and a 12-mm trocar was inserted at the port 4 position after sufficient dissection in the right retrorectus space. Retrorectus dissection was performed in the caudal direction, incising bilateral posterior rectus sheaths to the hernia defect (Fig. 3).

**Fig. 4:**
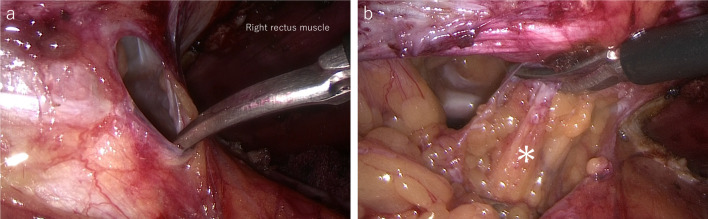
Intraoperative endoscopic views-2. Intraoperative endoscopic view. **a** Hernia sac was sharply dissected. **b** Mild adhesions between the hernia sac and omental tissue (*) were observed

**Fig. 5:**
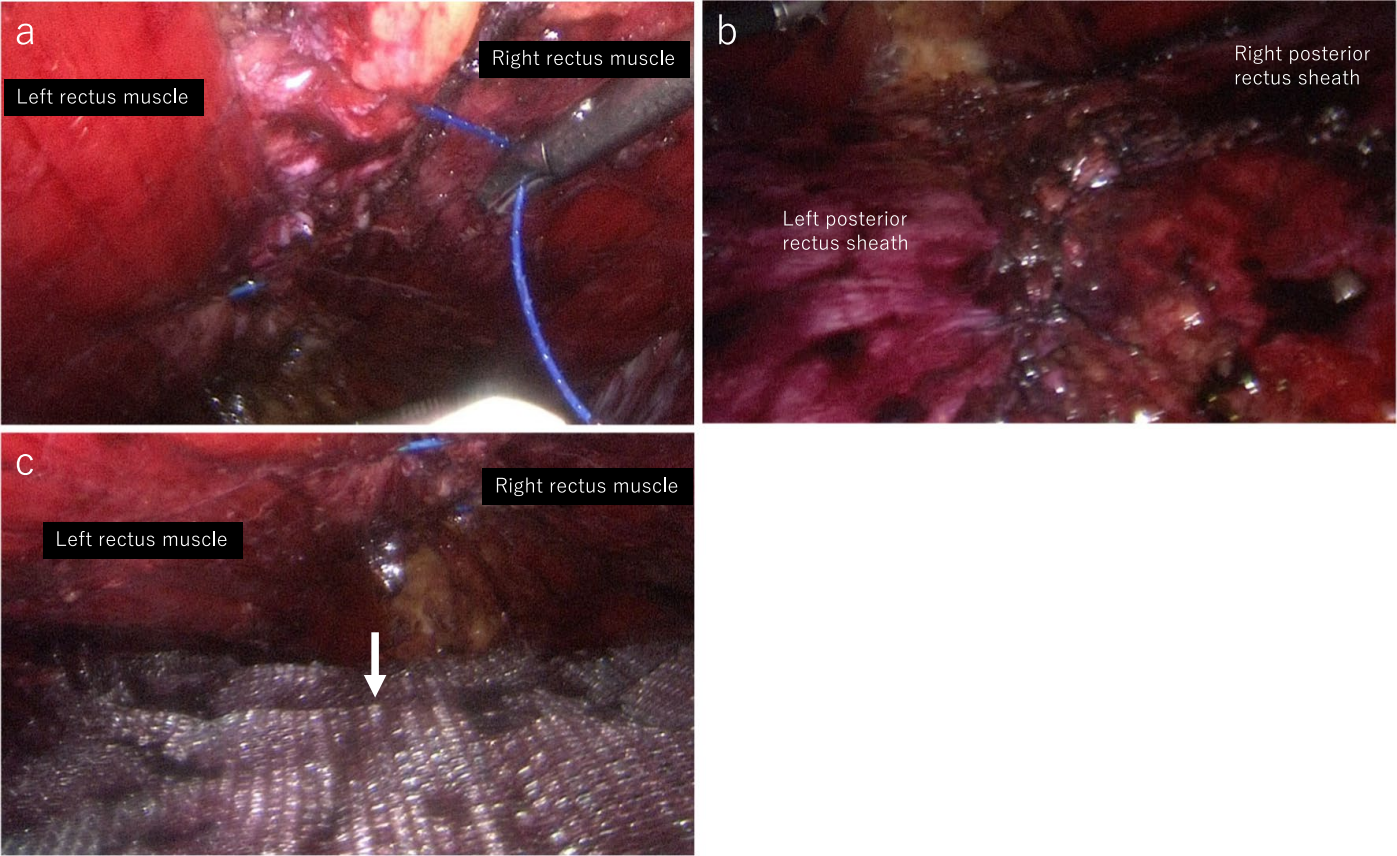
Intraoperative endoscopic views-3. **a** Abdominal defect was closed by 0 non-absorbable barbed suture. **b** Defect of the peritoneum was closed by 2–0 absorbable suture. **c** 20-cm-long and 15-cm-wide self-gripping mesh was placed in the retrorectus space with no fixation (arrow)

When the hernia sac was sharply dissected, we found very mild adhesions between the hernia sac and omental tissue (Fig. 4). Careful adhesiolysis was performed while confirming that there were no adhesions between the hernia sac and intestine. Bilateral retrorectus dissection was performed caudal to the arcuate line. After development of sufficient preperitoneal and bilateral retrorectus spaces, abdominal defect was closed by 0 non-absorbable barbed suture, and defect of the peritoneum was closed by 2–0 absorbable suture. Finally, a 20-cm-long and 15-cm-wide self-gripping mesh was placed in the retrorectus space with no fixation (Fig. 5). The operating time was 211 min, and the amount of bleeding was 5 g.

**Fig. 6:**
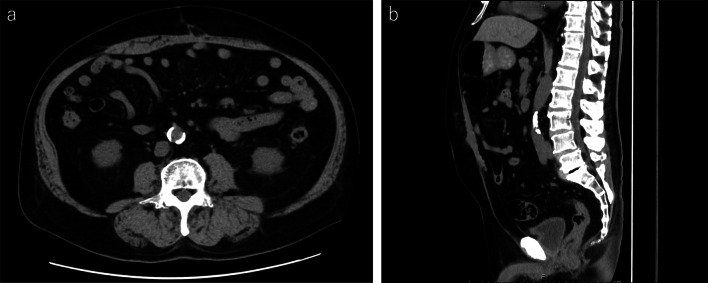
Post-operative abdominal computed tomography. Abdominal computed tomography at the time of 9 months after surgery shows no hernia recurrences. **a** Axial image. **b** Sagittal image

There were no postoperative complications and the patient was discharged on postoperative day 2. Postoperative pain was well controlled with prophylactic oral analgesics for 5 days after surgery. No hernia recurrences occurred after 9 months of follow-up (Fig. 6).

## Discussion

Recent large sample size cohort studies reported that the incisional hernia incidence rate was higher with minimally invasive radical prostatectomy, including RARP, than with traditional open radical prostatectomy [3, 4]. The incidence rate of incisional hernia following RARP has been reported to be 4.4–8.6% [8–11]. It is speculated that the incisional hernia occurrence rate after RARP was higher than that after open prostatectomy, because the camera trocar and specimen extraction site were placed above or in the umbilical region in RARP, where the muscles are known to be weaker, as opposed to the infraumbilical incision in open prostatectomy [4]. Given the recent worldwide spread of RARP for the treatment of prostate cancer, the number of incisional hernia cases after RARP is expected to increase.

The laparoscopic approach to incisional hernia repair with the IPOM technique was first described by LeBlanc in 1993 [12]. It has been reported that the laparoscopic approach to ventral hernia, including incisional hernia repair, has lower wound complication rates and faster recovery than the open approach [12, 13]. Therefore, laparoscopic repair of ventral hernia is rapidly becoming widespread, especially laparoscopic IPOM, the most popular technique [14]. However, the IPOM technique has serious complications, such as mesh-related adhesive bowel obstruction, mesh erosion, enterocutaneous fistula, and tacker-related chronic pain [15–17]. Postoperative pain has also been reported to be associated with double crown fixation and transfascial sutures [18].

The eTEP technique, which is an endoscopically performed Rives–Stoppa technique, was first described by Miserez for ventral hernia repair [6]. The eTEP can avoid the aforementioned complications by placing a mesh in the retrorectus space, not only eliminating contact between the mesh and intra-abdominal organs, which can avoid mesh-related complications, but also facilitating the minimization of using penetrating fixation, thereby avoiding tacker-related chronic pain as well [7, 19]. In a retrospective comparative analysis, Penchev et al. reported that the differences between eTEP and laparoscopic IPOM for repair of ventral hernias were the reduction in mean postoperative video analog scale pain score and the longer operative time, both in favor of eTEP. They considered that a lack of fixation in eTEP led to a reduction in postoperative pain [19].

When dissecting the hernia sac for eTEP repair, careful procedures are required to avoid injury to intra-abdominal organs, such as the small bowel. We consider that dissecting the hernia sac with a laparoscopic scissors in small steps is useful to avoid damaging the intra-abdominal organs. It is controversial whether a prosthesis can use for the incisional hernia repair if a bowel resection is performed as a result of a bowel injury [20, 21]. Although no studies have been reported on post-RARP adhesions, some studies have reported that laparoscopic surgery reduced adhesion formations for reasons, such as reduced peritoneal incision size, introduction of fewer foreign bodies, and less tissue trauma and bleeding than open surgery [22, 23]. Therefore, we consider that eTEP repair is one of the best treatment procedures for incisional hernia after minimally invasive surgery. On the other hand, we consider that eTEP repair is challenging for incisional hernia patients with large defects, for post-laparotomy cases, and for recurrence cases. For those challenging incisional hernia cases, endoscopic transversus abdominis muscle release, which is one of the posterior component separation procedures, is useful as a minimally invasive surgery [24]. Belyansky reported that transversus abdominis muscle release was helpful in cases with wide (> 10 cm) defect, tension of the posterior layer, narrow retrorectus space (< 5 cm), or when dealing with a compliant abdominal wall [7].

## Conclusion

eTEP repair is a very useful minimally invasive procedure for patients with incisional hernias after RARP. Although long-term follow-up is necessary to establish the safety and efficacy of eTEP repair, we consider this procedure to be the best option for patients with incisional hernia because of its few postoperative complications.

## Data Availability

The data sets supporting the conclusions of this article are included in this paper.

## Abbreviations

eTEP: Enhanced-view totally extraperitoneal
IPOM: Intraperitoneal onlay mesh
RARP: Robot-assisted laparoscopic radical prostatectomy
